# Significance of major international seaports in the distribution of murine typhus in Taiwan

**DOI:** 10.1371/journal.pntd.0005430

**Published:** 2017-03-06

**Authors:** Chi-Chien Kuo, Nicola Wardrop, Chung-Te Chang, Hsi-Chieh Wang, Peter M. Atkinson

**Affiliations:** 1 Department of Life Science, National Taiwan Normal University, Taipei, Taiwan; 2 Geography and Environment, University of Southampton, Southampton, United Kingdom; 3 Department of Geography, National Taiwan University, Taipei, Taiwan; 4 Center for Diagnostics and Vaccine Development, Centers for Disease Control, Ministry of Health and Welfare, Taipei, Taiwan; 5 Faculty of Science and Technology, Lancaster University, Lancaster, United Kingdom; 6 School of Geography, Archaeology and Palaeoecology, Queen's University Belfast, Belfast, Northern Ireland, United Kingdom; Fondation Raoul Follereau, FRANCE

## Abstract

**Background:**

International seaports are hotspots for disease invasion and pathogens can persist in seaports even after ports are abandoned. Transmitted by fleas infected by *Rickettsia typhi*, murine typhus, a largely neglected and easily misdiagnosed disease, is known to occur primarily in large seaports. However, the significance of seaports in the occurrence of murine typhus has never been validated quantitatively.

**Methodology/Principal findings:**

We studied the spatial distribution of murine typhus, a notifiable disease, in Taiwan. We investigated whether risk of infection was correlated with distance to international seaports and a collection of environmental and socioeconomic factors, using a Bayesian negative binomial conditionally autoregressive model, followed with geographically weighted regression. Seaports that are currently in use and those that operated in the 19^th^ century for trade with China, but were later abandoned due to siltation were analyzed. A total of 476 human cases of murine typhus were reported during 2000–2014 in the main island of Taiwan, with spatial clustering in districts in southwest and central-west Taiwan. A higher incidence rate (case/population) was associated with a smaller distance to currently in-use international seaports and lower rainfall and temperature, but was uncorrelated with distance to abandoned ports. Geographically weighted regression revealed a geographic heterogeneity in the importance of distance to in-use seaports near the four international seaports of Taiwan.

**Conclusions/Significance:**

Our study suggests that murine typhus is associated with international seaports, especially for those with large trading volume. Thus, one of the costs of global trade in Taiwan might be elevated risks of murine typhus. Globalization has accelerated the spread of infectious diseases, but the burden of disease varies geographically, with regions surrounding major international seaports warranting particular surveillance.

## Author summary

Globalization has hastened the spread of infectious diseases, with seaports as hotspots for disease invasion. Transmitted by fleas infected with the rickettsia *Rickettsia typhi*, murine typhus occurs worldwide, but its significance as a common causative agent of illness in tropical regions remains largely neglected. Although it is recognized that murine typhus is prevalent primarily in large seaports, the significance of seaports in the occurrence of murine typhus has never been validated quantitatively. We thus investigated whether distribution of murine typhus in Taiwan was associated with international seaports. Notably, abandoned international seaports (abandoned in the 19^th^ century due to siltation) were also studied to see whether the causative agent of murine typhus might still circulate around the ports even after being abandoned. We found that infection risk of murine typhus was negatively associated with distance to operating seaports but was uncorrelated with nearness to abandoned seaports. In addition, the importance of distance to operating seaports for risk of murine typhus infection varied spatially. Our study highlights elevated disease risk as a cost of international trade and suggests particular surveillance in regions surrounding major international seaports.

## Introduction

Trade is commonly accompanied by the spread of infectious diseases and international seaports have long been hotspots for disease invasion [1]. The great expansion in trade and international networks in recent history has seen seaports increasingly receive imported pathogens and vectors [2, 3]. For example, yellow fever has devastated seaports in the Americas due to the importation of the virus-infected mosquito *Aedes aegypti* (a competent vector for yellow fever) by ships [4]. Another new disease vector originating in Asia, *Aedes albopictus*, has also spread to seaports in both the Old and New Worlds [5, 6].

Successful introduction of exotic diseases involve arrival, establishment of local transmission, and subsequent spatial dispersal [7]. In suitable environments, exotic pathogens or parasites can persist in invaded regions even though these pathogens or parasites have ceased to arrive at the seaports. For instance, plague introduced to the USA through San Francisco in 1899–1900 still circulates among prairie dogs in the deserts of the Southwestern United Sates [8, 9] despite the absence of current importations. Likewise, helminths introduced by exotic rats have spread to indigenous mice on the California Channel Islands, with transmission persisting even after eradication of the rat hosts [10]. The probability of ongoing transmission following introduction to a new area is dependent on habitat suitability: for example, the availability of host species and/or vectors which may, in turn, be influenced by environmental conditions [11]. Thus, one legacy of past shipping events might be continuing circulation of exotic pathogens near receptive seaports; that is, although seaports have ceased to function, imported pathogens may persist in proximity to the abandoned seaports, if the conditions are suitable.

Murine typhus is a rickettsial disease with a worldwide distribution, but its significance as a common causative agent of illness in tropical regions remains largely neglected [12]. Transmitted by fleas infected with *Rickettsia typhi*, people typically acquire murine typhus via contaminated flea faeces near the bite sites instead of directly from the flea bites [13]. The life cycle of *R*. *typhi* commonly involves the oriental rat flea *Xenopsylla cheopis* and commensal rats, particularly *Rattus rattus* and *Rattus norvegicus* [14]. However, in suburban Southern California and Southern Texas, *R*. *typhi* is instead maintained by the cat flea *Ctenocephalides felis*, the opossum *Didelphis marsupialis* and domestic cats [15–18], and in Spain, dogs were found to host *R*. *typhi* [19]. It is well recognized that murine typhus is prevalent primarily in large seaports, probably due to the repeated introduction of infective fleas and rats [20]. Nevertheless, the significance of seaports in the occurrence of murine typhus has never been validated quantitatively. Likewise, while incidence of murine typhus is associated with the abundance of fleas, which is affected by climatic factors such as temperature, precipitation and humidity [20, 21], spatial analysis of the relationship between murine typhus and environmental variables remains very rare. The spatial distribution of murine typhus has been investigated in Lao PDR to confirm whether murine typhus is more common in urban areas, but only socio-economic risk factors have been included in the study [22]. Spatial clustering of murine typhus was also studied in Texas, but focusing on a comparison of clustering detection methods [23] instead of environmental correlates.

In Taiwan, murine typhus is an endemic disease, with 13 to 44 human cases annually during 2005–2014 (Taiwan Centers for Disease Control (CDC); http://nidss.cdc.gov.tw/). The spatial pattern of murine typhus occurrence and the reasons for geographic heterogeneity have never been explored in Taiwan; instead, past studies have focused on clinical manifestations of the disease [24–28]. We conducted a retrospective investigation of the spatial distribution of murine typhus in Taiwan and explored its association with environmental and socioeconomic factors. Notably, we sought to determine whether murine typhus incidence was higher in areas closer to international seaports. Seaports that are currently in use and abandoned seaports were analyzed to identify the public health consequences of historical international trade. Occurrence of murine typhus could also be related to the presence of cats, dogs and cat fleas, as recently found in Spain and the United States of America [16, 19]. However, the lack of information on the number of cats and dogs (particularly stray ones) and the spatial distribution of cat fleas in Taiwan hindered incorporation of this non-classic infection route in this research. The current study therefore focused on the classic rat-flea transmission cycle, which remains the primary route of infection all over the world [15].

## Methods

### Ethical statement

The case records were retrieved from the Taiwan National Infectious Disease Statistics System administrated by Taiwan Centers for Disease Control (Taiwan CDC) and no personally identifiable information were used as part of this study.

### Study area

This study focused on the main island of Taiwan. Small associated islets were excluded (Kin-men, Ma-tou, Peng-hu, Little Liu-chiu, Ci-jin, Green, and Orchard islands) because they frequently differ with regard to potentially important ecological characteristics (e.g., animal communities [29]). The basic geographical units used in this analysis were administrative districts (within urban cities) and townships (within rural counties); these are the smallest administrative areas to which murine typhus cases can be assigned. In this study, we use “district” to refer to both the urban districts and the rural townships.

### Disease incidence

Human incidence of murine typhus from 2000 to 2014 was retrospectively analyzed in this study. Murine typhus is a notifiable disease in Taiwan. Blood samples from patients with suspected murine typhus are collected and sent to the Taiwan CDC for laboratory diagnosis. Samples were considered positive for murine typhus based on a positive real-time polymerase chain reaction (PCR) test or the detection of *R*. *typhi*-specific antibodies based on the indirect immunofluorescent assay (IFA). The real-time PCR test targeted the 17-kDa antigen in *Rickettsia* spp. and the PCR products were sequenced and then assessed with the Basic Local Alignment Search Tool (www.ncbi.nlm.nih.gov) for resemblance to known *Rickettsia* spp. For IFA, each serum sample was applied to slides coated with *R*. *typhi* antigens (Focus Technologies, Inc., Cypress, CA, U.S.A.). Two IFA criteria were applied: (1) four-fold increase in *R*. *typhi*-specific immunoglobulin M (IgM) or IgG antibody in paired sera (each for the acute and convalescent phase, with interval >14 days); (2) positive for patient with IgM 1:80 dilution and IgG 1:320 dilution. Because infection may occur away from a patient’s residence, starting in 2003, the presumptive location of infection was recorded as well as the patient’s residence. These data, along with gender, age, and date of symptom onset, are available from the Taiwan CDC. To more accurately assess the relationship between infection and environmental factors, we allocated cases of murine typhus (2003–2014) to the presumed district in which the infection occurred rather than the district in which the patient resided. For incidences during 2000–2002, patient’s residence was used instead. The presumed district of infection and district of residence were the same for 97.1% of cases from 2003 to 2014, so the use of patient’s residence from 2000 to 2002 is not considered problematic. Because yearly variation (2000–2014) in district population size was low (3.7%, average value of (standard deviation divided by mean) for all districts), population size for each district was represented by the mean value from 2000–2014. Population size was obtained from the Department of Statistics of the Taiwan Ministry of the Interior (http://sowf.moi.gov.tw/stat/month/list.htm), and the murine typhus incidence rate (IR, number of cases per 100,000 people per year) was calculated for inter-district comparisons.

### Spatial clusters of murine typhus incidence

The presence of spatial autocorrelation of the murine typhus IR (incidence rate) was assessed using Moran’s I [30]. The locations of spatial clusters of murine typhus incidence were identified using local indicators of spatial association (LISAs). LISAs can be treated as a local version of Moran’s I [30], and can be used to detect local clusters of observations with similar or dissimilar values [31]. A map of LISAs clusters, thus, allowed the assignment of each district to one of five categories: high-high, which indicates a district with high IR surrounded by districts with high IR (also called a hot spot); low-low, a district with low IR surrounded by low-IR districts (a cold spot); low-high or high-low, a district with low IR surrounded by high-IR neighbors and *vice versa*; and not significant, which indicates a district with no significant local autocorrelation [32]. Inference for significance of Moran’s I and LISAs was based on 99,999 permutations using the GeoDa 0.9.5 software [33], and empirical Bayes (EB) was applied to correct for large variation in population size among districts [32], with population size as the base variable. The threshold of significance was set at *P* = 0.05, and maps were displayed using QGIS 2.12 (QGIS Development Team).

### Environmental and socioeconomic variables

We selected variables for analysis based on the availability of data and our knowledge of the study system. Twelve explanatory variables (seven environmental variables, two socioeconomic variables, and three port distance variables) were included in the study. Environmental variables included elevation (elevation, meters), total annual rainfall (rainfall, mm), mean annual temperature (temperature, °C; calculated as the mean of 12 monthly mean temperatures), number of days with temperature higher than 30°C within a year (daysT30), relative humidity (%) and a selected list of land cover categories. Elevation was derived from a 40-m digital elevation model (Aerial Survey Office of Taiwan Forestry Bureau). The four meteorological variables were obtained from Central Weather Bureau of Taiwan (*n* = 390 meteorological stations, the Data Bank for Atmospheric Research is available at https://dbahr.narlabs.org.tw/) and were calculated over the period 1991 to 2013. The spatial layers of climatic variables were generated at a spatial resolution of 1 km by interpolation (390 stations) using Kriging in ArcGIS with a spherical variogram model [34]. We overlaid administrative district boundaries and calculated the mean values for elevation, rainfall, temperature, days over 30°C and relative humidity for each district. Land cover data were obtained from the Globcover database [35] using a spatial resolution of 30 arc seconds (ca. 1 km) and the initial land cover classes were merged to create a smaller number of land cover types likely to be important for *R*. *typhi* transmission. These include artificial structure and forests (artificial surface and forest) because human infection of *R*. *typhi* occurs mainly inside buildings [20] and we were interested in the potentially protective effects of forests. The proportion of each district that consisted of each of these land cover classes was calculated to provide a quantitative characterisation of the land cover.

To assess the role of socioeconomic factors, average income (income) of each district for the year 2005 was obtained from the Fiscal Information Agency of the Taiwan Ministry of Finance (http://www.fia.gov.tw/). Population density for each district was obtained by dividing population size by the respective administrative area.

### Distance to international seaports

In this study, distance to three different types of international seaports were analyzed for comparison with *R*. *typhi* infection: (1) currently in use (n = 4); (2) operated mainly during the 19^th^ century for the trade of commodities with mainland China, where murine typhus has long been prevalent along the coast [36], but were largely abandoned later because of siltation (*n* = 26); and (3) including both in-use and abandoned international seaports (*n* = 28). Two seaports which were operational during the 19^th^ century remain in operation today, and so are included in all three of these categories.

**Fig 1:**
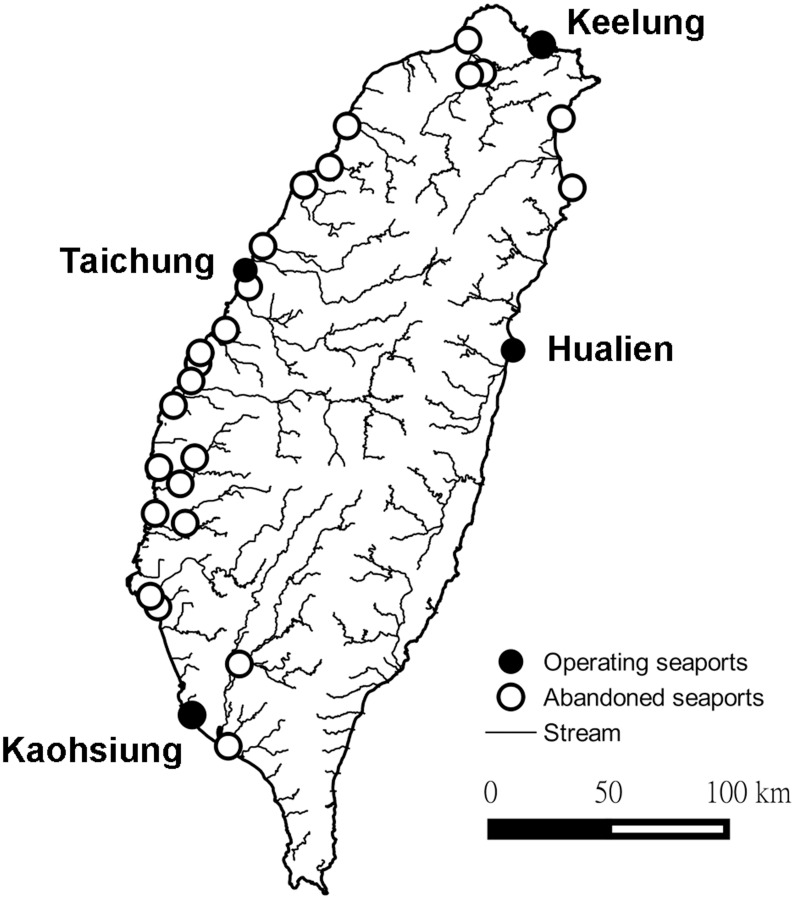
International seaports that are currently in use and those that operated in the 19^th^ century for trade with China but was abandoned since late 19^th^ century.

International seaports that are currently operated include Keelung, Taichung, Kaohsiung, and Hualien seaports (Fig 1). Keelung and Kaohsiung seaports have been in use since the 19^th^ century while Taichung and Hualien seaports have been operated since the 1970s. 19^th^ century Taiwanese seaports have been classified into ten categories based on the volume of seaborne goods handled [37]. Some of the ports with the largest amount of cargo handled were deemed international ports in this study, because each had direct marine traffic with mainland China [37]. These 26 international ports are mostly located along the coast although some are situated along rivers (Fig 1) and only two of them (Keelung and Kaohsiung) continue to engage in international trade. Distance to international seaports was represented by the Euclidean distance from the geographical centroid of each district to the nearest ports. Overlay of the district boundaries on grids of environmental variables and the calculation of the nearest distance to ports were implemented in ArcGIS 10.2.

### Relationship between incidence and explanatory variables

Correlation analysis was applied to assess the strength and direction of the association amongst the explanatory variables. Where variables were highly correlated with one another, only one of the variables was retained for subsequent non-spatial multivariate regression analysis to avoid multi-collinearity. Lastly, significant variables in the final non-spatial multivariate model were analyzed separately with a Bayesian spatial regression model and geographically weighted regression.

#### Non-spatial univariate negative binomial regression

Firstly, pair-wise Spearman’s rank correlations were applied to the twelve explanatory variables (seven environmental variables, two socioeconomic variables, and three port distance variables) to assess collinearity. Where two (or more) variables were highly correlated with one another, (*r*_*s*_ >0.7; [38, 39]) we removed all but one, retaining the variable with the most statistically significant *z* value using a univariate negative binomial regression. Negative binomial regression was applied due to over-dispersion in the murine typhus case data in this study; the count of murine typhus cases was the response variable and the logarithm of human population size was used as an offset to control for its potential influence on disease incidence. Non-significant variables (*P*>0.05) in the univariate model were also removed from further multivariate analyses.

#### Non-spatial multivariate negative binomial regression

All explanatory variables, except those removed during the previous step due to non-significance or high collinearity, were then analyzed using a multivariate negative binomial regression. The least significant variable (with the highest *P* value) was subsequently removed from the model until all retained variables were statistically significant. Residuals of the final multivariate negative binomial model were assessed for spatial correlation using Moran’s *I* with 99,999 permutations in the GeoDa 0.9.5 software [33].

#### Bayesian negative binomial Conditionally Autoregressive (CAR) model

A conditionally autoregressive (CAR) prior was incorporated in the multivariate negative binomial model to address spatial autocorrelation in the residuals, as revealed by Moran’s *I* (see Results). The CAR component accounts for spatial dependency by modeling the residual of one observation as a function of neighboring residual terms [40]. Adapted from [41], the negative binomial CAR model can be represented as:

$$\text{log }\mu_{i}=\text{ log }E_{i}+\theta_{i}$$

$$y_{i}\sim \text{ NB }(p_{i}{}_{,}r)$$

$$p_{i}=r/\;(r+\mu_{i})$$

where *y*_*i*_, following a negative binomial distribution, denotes counts of cases of murine typhus for district *i* (*i* = 1 to 349) with a mean of *μ*_*i*_ and shape parameter *r*. *E*_*i*_, the expected number of cases of murine typhus within district *i*, is an offset term used to control for population size within a district. *θ*_*i*_ is the log relative risk. The log relative risk *θ*_*i*_ was modeled as:

$$\theta_{i}=\beta_{0}+\sum_{J=1}^{p-1}\left(\beta_{j}x_{j}\right)+S_{i}$$

where *β*_*0*_ is the intercept, *β*_*j*_ is the coefficient for explanatory variable *x*_*j*_, and *S*_*i*_ is the CAR component which accounts for spatial correlation in the residuals of neighboring districts. Districts with shared borders, including shared corners, are defined as neighborhoods (i.e., queen adjacency). Each neighboring district was assigned a weight of 1, and 0 otherwise.

The estimation of coefficients of parameters were based on Bayesian inference using a Markov chain Monte Carlo (MCMC) algorithm and Gibbs sampling method. A non-informative, flat prior was assigned to *α* and normally distributed priors, with mean zero and precision 0.01, were assigned to *β*_1*…j*_. Following an initial burn-in period of 10,000 iterations, a further 300,000 iterations were performed, and every tenth iteration was stored for parameter estimation, to reduce autocorrelation in the samples. MCMC chains were inspected to check for convergence and ensure that the initial burn-in period was long enough to avoid autocorrelation. Besides, multiple initial values for each parameter were used to ensure the MCMC algorithms were converging on the same parameter space from different starting points. Monte Carlo error, a measure of Bayes sampling error, was confirmed to be <5% of the posterior standard deviation for each parameter [42]. Summary measures for the posterior distribution of each parameter (posterior mean, standard deviation and 95% credible interval (CrI)) were stored to provide parameter estimates. Based on the final Bayesian model, a smoothed relative risk map of murine typhus that incorporated information on fixed and random effects was created and displayed using QGIS 2.12 (QGIS Development Team). All statistical analysis was implemented in R 3.1.0 (R Core Team).

#### Geographically weighted regression model

Relative to the global model (e.g. the Bayesian CAR model as presented in the preceding section) where coefficients are spatially uniform across districts, geographically weighted regression allows the intercept and coefficients of explanatory variables to vary with district *i*:

$$y_{i}=\beta_{i0}+\sum_{j=1}^{p-1}\left(\beta_{ij}x_{ij}\right)+\varepsilon_{i}$$

Centered on each district, a moving window with pre-determined bandwidth and spatial weighting kernel function allows estimation of the intercept and coefficients for explanatory variables for each centered district, based on information provided within the window boundary. The bandwidth was determined using the adaptive golden section search method [43] based on the Akaike information criterion (AICc, with a correction for finite sample sizes). Among the four kernel types (fixed Gaussian, adaptive Gaussian, fixed bi-square and adaptive bi-square), the one with the lowest resultant model AICc value was selected as the kernel function in the weighted regression model. The count of murine typhus cases in each district was fitted with a multivariate Poisson model to assess its association with environmental and socioeconomic variables, with the logarithm of human population size used as an offset to control for its influence on disease cases. Only significant explanatory variables based on the Bayesian CAR model were included in the analysis, and significance (*P*<0.05) was defined as pseudo *t* value >1.96 or <-1.96 [43]. The analysis was implemented in GWR4.0 (GWR4 Development Team) and the map was displayed with QGIS 2.12 (QGIS Development Team).

## Results

### Disease incidence of murine typhus

**Fig 2:**
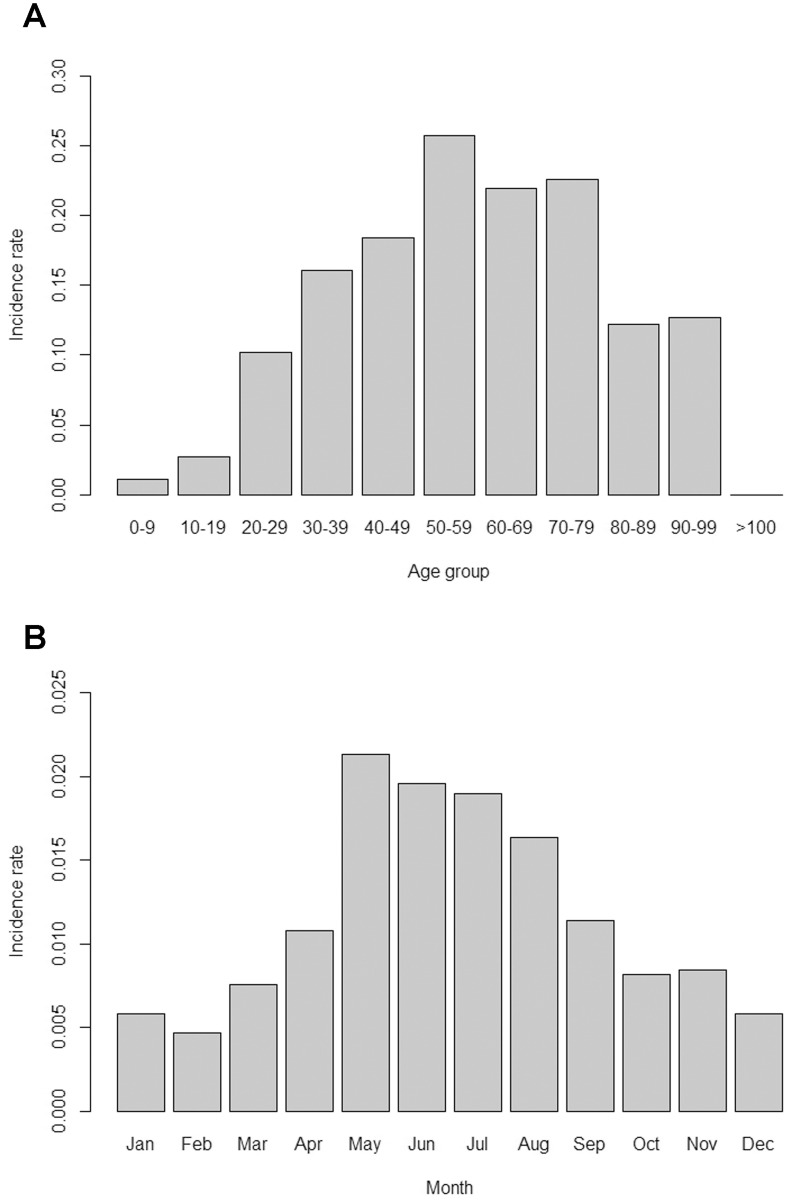
Variation in incidence rate (cases per 100000 people per year) of murine typhus among (A) age groups and (B) months in Taiwan during 2000–2014.

A total of 476 human cases of murine typhus were recorded during 2000–2014, with an incidence rate of 0.14 cases per 100,000 residents per year; this was higher in males than in females (0.20 vs. 0.08; Chi-square test with Yates’ correction, χ^2^ = 85.0, *P* < 0.001). The incidence rate also varied with age (χ^2^ = 167.1, *P* < 0.001) and was higher in the 50–79 age range (Fig 2A). There was also a significant seasonal variation in incidence rate (χ^2^ = 114.6, *P* < 0.001), with rates higher in later spring and summer than in other seasons (Fig 2B).

### Spatial distribution of murine typhus incidence

**Fig 3:**
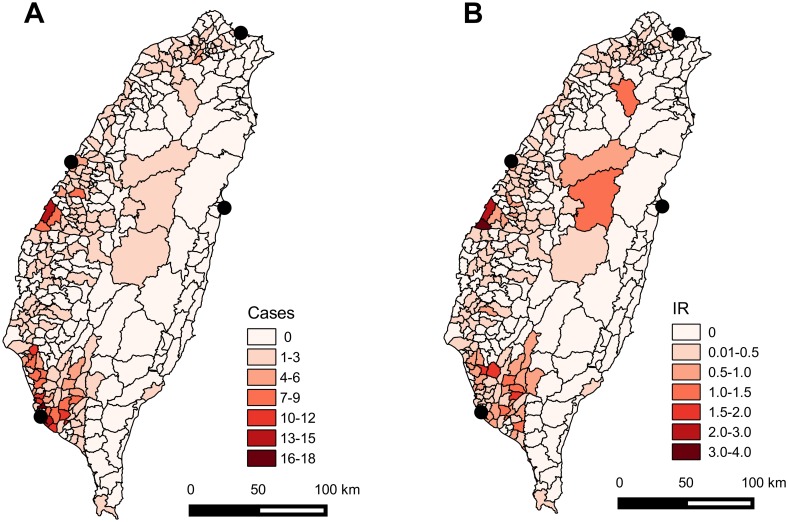
Spatial variation in (A) cases and (B) incidence rate (cases per 100000 people per year) of murine typhus among districts in Taiwan during 2000–2014. Black circles denote international seaports that are currently in use.

Among the 349 districts, the number of cases of murine typhus during 2000–2014 ranged from zero to 16 cases, with more cases occurring in southwest and central-west Taiwan (Fig 3A). The IR varied from zero to 3.1 cases per 100,000 residents per year and was higher in southwest and central-west Taiwan, along with central Taiwan (Fig 3B).

**Fig 4:**
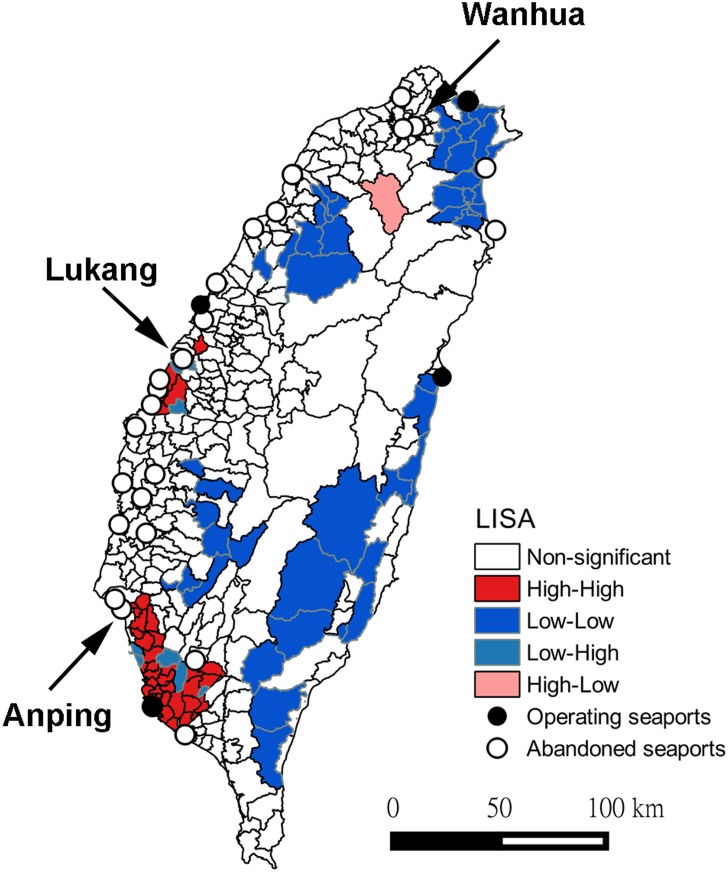
LISA (Local Indicators of Spatial Association) map of murine typhus in Taiwan during 2000–2014 and spatial association with international seaports. See text for definition of map legend. The threshold of significance was set at *P* = 0.05. Wanhua, Lukang and Anping were regarded as the largest ports in 19^th^ century in Taiwan.

Incidence of murine typhus was not randomly distributed in Taiwan (Moran’s *I* = 0.35, *P*<0.0001). Instead, the LISA map revealed that hot spots were present in southwest and central-west Taiwan while cold spots occurred in eastern Taiwan (Fig 4).

### Relationship between incidence and explanatory variables

#### Univariate negative binomial model

Univariate negative binomial regression showed that two determinants, income and population density, were insignificantly associated with the murine typhus IR (*P*>0.05, Table 1). In addition, elevation, daysT30, relative humidity, and distance to operating or abandoned international ports were dropped from further multivariate analyses due to collinearity (*r*_*s*_ >0.7) with other more significant variables (Table 1).

**Table 1:** Association of murine typhus incidence rate in districts in Taiwan during 2000–2014 with environmental and socioeconomic variables and distances to seaports, from univariate negative binomial regression.

Variable	Coefficient	*z* score	*P* value
Elevation (1000 m increase)*	-1.27	-3.146	<0.005
Rainfall (1000 mm increase)	-0.80	-5.149	<0.001
Temperature (1°C increase)	0.45	7.694	<0.001
DaysT30 (1 day increase)*	0.02	7.3	<0.001
Relative humidity (1% increase)*	-0.31	-6.585	<0.001
Artificial surface (1% increase)	0.69	2.237	0.03
Forest (1% increase)	-2.40	-5.97	<0.001
Income (1000 New Taiwan dollars increase)	0.16	0.266	0.79
Population density (1000 people/ha increase)	0.81	0.575	0.57
Distance to operating ports (10 km increase)	-0.21	-5.848	<0.001
Distance to abandoned ports (10 km increase)	-0.29	-4.433	<0.001
Distance to operating or abandoned ports (10 km increase)*	-0.30	-3.81	<0.001

* dropped in further multivariate analyses due to correlation (*r*_*s*_ >0.7) with other more statistically significant explanatory variable

#### Non-spatial multivariate negative binomial model

Six variables (rainfall, temperature, artificial surface, forest, distance to operating ports, and distance to abandoned sports) were included in the non-spatial multivariate negative binomial regression model. Artificial surface, forest and distance to abandoned seaports were removed from the final non-spatial model as they were not significantly associated with the IR (*P*>0.05). Of the three remaining variables, temperature was positively associated (per 1°C increase, *r* = 0.30, *P*<0.001), while rainfall and distance to operating ports were negatively associated (per 1000 mm increase, *r* = -0.51, *P*<0.005; per 10 km increase, *r* = -0.15, *P*<0.001, respectively), with IR. Residuals of the non-spatial multivariate model were not randomly distributed based on Moran’s *I*, necessitating the use of a spatial term in the regression model (Moran’s *I* = 0.24, *P*<0.0001).

#### Bayesian negative binomial CAR model

**Fig 5:**
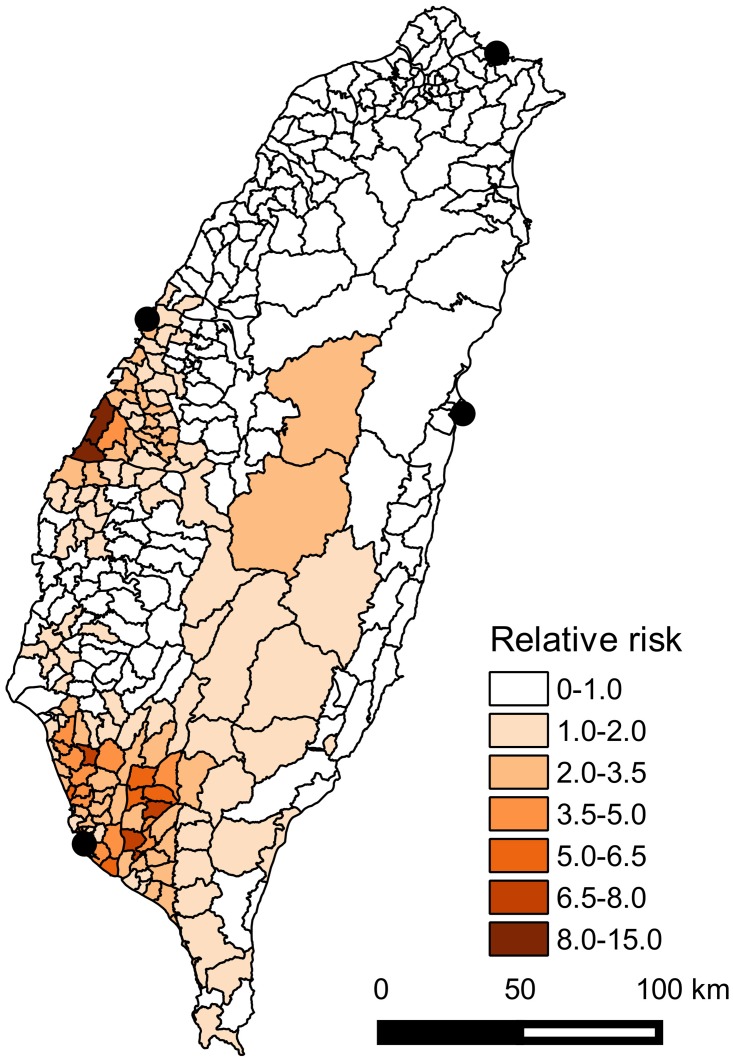
Relative risk map of murine typhus in Taiwan during 2000–2014 after incorporating fixed and random effects of Bayesian negative binomial CAR model.

A Bayesian negative binomial CAR model, which corrected for spatial dependency in the residuals, showed that temperature (posterior mean = -0.75 per 1000mm increase, 95% CrI = -1.4 to -0.1), rainfall (mean = -0.24 per 1°C increase, 95% CrI = -0.4 to -0.05) and distance to operating ports (mean = -0.21 per 10km increase, 95% CrI = -0.4 to -0.05) were significantly and negatively associated with murine typhus IR (95% CrI did not cross 0). The relative risk map that accounted for fixed (temperature, rainfall and distance to operating seaports) and random (spatially correlated and non-spatial) effects showed a higher risk of murine typhus primarily in southwest and central-west Taiwan (Fig 5). In comparison, northern and eastern Taiwan had relatively lower risk (Fig 5).

#### Geographically weighted regression model

**Fig 6:**
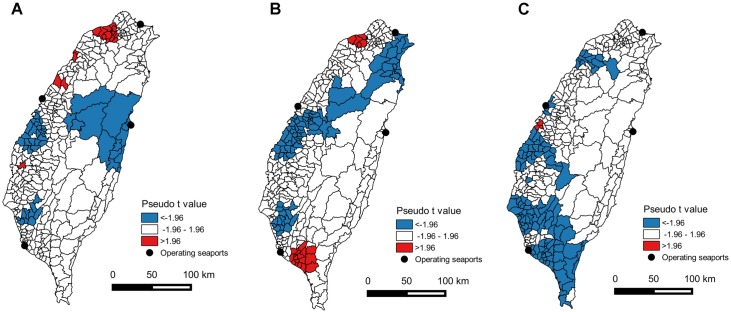
Geographically weighted regression pseudo *t* values for explanatory variable (A) temperature, (B) rainfall and (C) distance to operating ports. Pseudo *t* values larger than 1.96 or smaller than -1.96 were deemed significant (*P*<0.05). Positive and negative pseudo *t* values represented positive and negative association with murine IR, respectively.

The adaptive bi-square kernel was selected because it resulted in the lowest AICc value (547.2) compared to the other three kernel types (fixed Gaussian: 562.4, adaptive Gaussian: 604.9, fixed bi-square: 589.4). The statistical significance of temperature, rainfall and distance to operating ports in the spatial distribution of murine typhus cases varied considerably between districts. Temperature was negatively associated with murine typhus IR (pseudo *t* <-1.96, *P*<0.05) mainly in central-east, central-west and southwest Taiwan, but there were also a few districts in northwest Taiwan with a positive correlation (pseudo *t* >1.96; Fig 6A). A negative association between rainfall and murine typhus IR occurred mainly in northeast, central-west and southwest Taiwan; by contrast, some districts in northwest and southwest Taiwan revealed a positive correlation (Fig 6B). Lastly, distance to operating ports was negatively related to murine typhus IR, primarily in districts surrounding Kaohsiung port and to a lesser degree near Taichung port although there were also two districts close to Taichung port that instead exhibited a positive association (Fig 6C).

## Discussion

This research has examined the spatial distribution of murine typhus in Taiwan, and possible explanatory factors for this distribution, for the first time. We found spatial clustering of human cases of murine typhus in southwest and central-west Taiwan. The risk of infection was higher in areas closer to international seaports that are currently in use, particularly near Kaohsiung and Taichung seaports. However, the probability of infection was not significantly associated with distance to *abandoned* international seaports. Risk of murine typhus was also negatively associated with rainfall and temperature, after controlling for distance to in-use international seaports.

It has been stated that ports are the primary *foci* of murine typhus transmission [20]. Nevertheless, to the best of our knowledge, this is the first study to quantitatively validate a negative association between risks of *R*. *typhi* infection and distance to seaports, based on an advanced spatial modeling approach. Higher risks of infection near (active) seaports suggest that these may be the source of infection, as a consequence of repeated introduction of infective rats and/or fleas from abroad in combination with the mild weather typically enjoyed by coastal cities that is also hospitable for rats and fleas [20]. In spite of the negative association between disease incidence and distance to operating international seaports, the IR of murine typhus and the importance of distance varies considerably among the four ports. Distinctly, negative association between IR of murine typhus and distance to operating seaports occurs primarily near the Kaohsiung and Taichung seaports (Fig 6C). There have been no cases surrounding the Hualien seaport in eastern Taiwan and there are very few cases along the eastern coast of Taiwan (Fig 3), in stark contrast with the high prevalence along the western coast, particularly near the Kaohsiung and Taichung seaports. This is consistent with the finding of a higher seropositivity rate of *R*. *typhi* infection in shrews and rodents trapped in Kaohsiung seaport (26.1%) and Taichung seaport (18.1%) than the other eight seaports or airports (including Keelung seaport of 0.7% and Hualien seaport of 1.7%, [44]). Such geographical variation could be due to the remarkable difference in trading volume among the four ports, with Kaohsiung dealing with the lion’s share of international cargo (an annual mean of 112 million tons during 2011–2013), followed by Taichung (60 million tons), Keelung (18 million tons), and Hualien (4 million tons) (Taiwan International Ports Corporation; http://www.twport.com.tw/). The lack of cases in proximity to Hualien might be related to the smaller cargo volumes providing fewer opportunities for pathogen introduction although this could also be related to higher temperature and rainfall near this port (Supporting information S1 Fig) so that pathogen transmission cannot be easily sustained after being imported. It was also found that the spatial distribution of murine typhus differed from that of scrub typhus, another rickettsial disease transmitted by mites. In Lao PDR, murine typhus was more common in urban areas while scrub typhus was more common in rural regions [22]. This contrasting spatial distribution also occurs in Taiwan, where scrub typhus is much more prevalent in less developed eastern areas than industrialized western areas of Taiwan [45, 46]. Although *R*. *typhi* was not detected in fleas in eastern Taiwan [47], rickettsial strains similar to *R*. *typhi* have been detected in ticks and rodents in the same region [48, 49], indicating that murine typhus might also circulate in this region but may be overlooked by physicians. This could be due to low prevalence as revealed by the low seropositivity rate of *R*. *typhi* infection in shrews and rodents in Hualien seaport (1.7%, [44]). Our study suggests that murine typhus should be considered as a possible diagnosis when patients close to the Hualien seaport present with suspected rickettsial infections. Indeed, clinical manifestations of many rickettsial diseases (e.g. high fever, headache, rash) are so similar that identification of the etiologic agent is very challenging, especially in the tropics [50]. Under-reporting is thus likely to be common, particularly in Hua-lien, where the other rickettsial disease (scrub typhus) is very prevalent [45, 46] and murine typhus might be readily excluded.

Although it is expected that poorer hygiene in the 19^th^ century vessels might render rats infested with fleas more likely to board ships and invade ports, we did not find quantitative evidence supporting higher risks of infections near ports that operated in the 19^th^ century, but which have subsequently been abandoned. This suggests that local conditions might not be suitable for long-term sustained transmission, and as these ports have largely been abandoned since the late 19^th^ century, there was little opportunity for recent introduction at these locations. Because more people work in operational than abandoned seaports, more food might be available to sustain a higher population of rats and fleas in operational than abandoned ports. However, whether the lower risks were the result of lower survival of rats, fleas or *R*. *typhi* in abandoned ports remains to be investigated due to a lack of systematic studies on these ports. Another possibility is that the transmission cycle is sustained to the current date in so few abandoned ports that the significance of abandoned ports cannot be established statistically. In other words, contemporary infection might continue in a few abandoned ports since the 19^th^ century, but because infection has ceased in most abandoned ports, we were unable to recognize its significance when all obsolete ports are considered in spatial analysis. For example, while the two hotspots in southwest and central-west Taiwan are also close to abandoned seaports (Fig 4), the majority of obsolete ports have very low incidence; although a similar spatial pattern could also arise where there are so many abandoned ports that hotspots coincidently occur close to a few of them.

It is very difficult to unpick the significance of in-use versus abandoned seaports although our results suggest that some in-use seaports are of more importance for contemporary murine typhus incidence than abandoned ports. One potential solution to this issue is to investigate the population genetic structure of *R*. *typhi* in Taiwan. Cargos moving through abandoned and operating seaports came from different locations: abandoned seaports are likely to have dealt with cargo mainly from coastal China, while in-use seaports are likely to deal with cargo mainly from other countries. Therefore, the genetic composition of *R*. *typhi* should differ based on origin prior to introduction to Taiwan. This information would allow a more comprehensive assessment of the importance of abandoned seaports in the contemporary spatial distribution of infection. Studying the population genetic structure would also help discern whether *R*. *typhi* is mostly imported (i.e. genetic composition differs among international seaports) or is spread from within Taiwan (i.e. no spatial structure in genetic composition is observed). The current status of rats and fleas (species and abundance) in operational and abandoned ports could also be better understood when trapping rodents to investigate the genetic structure of *R*. *typhi* in fleas; this could help reveal how the non-sustained transmission of *R*. *typhi* in abandoned ports could be related to the survival of rats or fleas. Another limitation of the current study is that due to the lack of data on trading volume at abandoned seaports [37], the probability of importation of *R*. *typhi* at each seaport is considered identical, even though the volume of trade varies considerably among ports. Anping, Lukang and Wanhua were regarded as the largest ports in the 19^th^ century in Taiwan, but there was no evidence of spatial clustering around these obsolete ports (Fig 4), suggesting that historical trading volume might not be the primary determinant of contemporary murine typhus infection risks.

Lastly, whereas serological assay is the primary method for diagnosis of murine typhus [51], cross-reactivity can occur in human sera against *R*. *typhi* and *R*. *felis* antigens [52–55], although it is unclear why similar cross-reactivity does not always occur [e.g. 56, 57]. Potential serologic cross-reactivity suggests that confirmed cases of murine typhus based solely on IFA diagnosis may include some misdiagnosed *R*. *felis* infections (also a flea-borne rickettsial disease). In Taiwan, molecular methods have detected *R*. *felis* or *R*. *felis*-like organisms in one patient [58] as well as in small mammals [49] and fleas [47, 59, 60]. Therefore, we cannot exclude the possibility that murine typhus cases confirmed by the Taiwan CDC may include some cases of *R*. *felis* and the case data might more accurately reflect infections of flea-borne rickettsial diseases (caused by *R*. *typhi* or *R*. *felis*) instead of infectious associated with *R*. *typhi* only. In Taiwan, however, sera of confirmed cases of murine typhus were not found to cross-react with *R*. *felis* antigen [61] and serum from the single patient detected with *R*. *felis* nucleotides did not cross-react with *R*. *typhi* antigen [58]. Based on this, the extent of *R*. *felis* infections in patients diagnosed with murine typhus is presumed to be minimal in Taiwan, but this warrants further investigation. Also awaiting validation is the importance of *R*. *felis* as the causative agent of human illness, which is recently questioned for its widespread distribution in cat fleas but few and spatially restricted human cases of flea-borne rickettsioses in California [62, 63] and its ubiquity in a diverse array of arthropods and also in healthy people in Africa [51]. Whether *R*. *felis* is simply a symbiont of arthropods similar to *Wolbachia* [64] would therefore determine if infection of *R*. *felis* is required to be considered in epidemiological studies of murine typhus.

Across Taiwan, rainfall and temperature were also significantly associated with murine typhus incidence, after controlling for the influence of distance to operating international seaports. Given the same distance to seaports, murine typhus incidence decreased with increasing rainfall and temperature. This suggests that after *R*. *typhi* was introduced at the ports, the probability of further inland invasion of rats, fleas or *R*. *typhi* may have been determined by local climates. It was found that in eastern Taiwan, fleas were more abundant in months with less rainfall and lower temperature, but the underlying mechanism still awaits investigation [47]. In fact, climatic effects on flea-borne diseases are complex and context dependent. For example, similar to murine typhus, transmission of plague also involves bacteria, fleas, and rodent hosts. While it is generally thought that fleas prosper under hot and humid conditions, *Yersinia pestis*, the etiologic agent of plague, can persist in arid regions (such as Central Asia and Western USA), but is less likely to sustain transmission in humid tropical areas [21]. Likewise, fleas were predominantly collected in dry rather than humid regions. However, in the dry part of Reunion Island, fleas were more abundant during the hot-wet season [65]. This is akin to our finding that geographically, murine typhus tended to occur in cooler and drier areas, but seasonally murine typhus was more prevalent in the warmer season (late spring and summer). Apart from fleas, climate could also influence the abundance of rodents and human behavior [21], both of which could affect the infection risk of murine typhus. The precise relationship between rainfall, temperature and murine typhus incidence in Taiwan (given the same distance to seaports) is, therefore, complex and necessitates great care to disentangle it. This is further supported by the geographical heterogeneity in the importance and direction of relationship for temperature and rainfall (Fig 6A and 6B). Moreover, it should be emphasized that while an association between infection risk of murine typhus and climatic variables is identified, such correlation does not definitely represent that climate does determine the risk of infection; other not-recognized variables correlated with rainfall and temperature might instead be the main determinant.

Our study demonstrates that one of the costs of international trade in Taiwan might be an elevated risk of murine typhus. This can be exemplified by Kaohsiung seaport, whose container traffic ranks 13^th^ globally (World Shipping Council; www.worldshipping.org). Kaohsiung is not only a hotspot for murine typhus (this study); dengue and scrub typhus, both vector-borne diseases, are also common in this port city [45, 66]. To prevent potential importation of exotic diseases, regulations mandated by Taiwan CDC that govern quarantine at international ports require all arriving ships to report occurrence of rodents and disease vectors on the ships. Small mammals, fleas and seroprevalence of *R*. *typhi* in rodents are also monitored constantly and the eradication of rats has been attempted in these four international seaports by Taiwan CDC [44]. Globalization has hastened the spread of infectious diseases [67, 68], but the burden of diseases varies geographically, and as this study has shown, regions surrounding international seaports should warrant particular surveillance. Also needed is the assessment of whether eradication programmes implemented in seaports do indeed mitigate the risks of targeted diseases.

## Supporting information

S1 Fig Spatial variation in (A) annual mean temperature (°C) and (B) total annual rainfall (mm) among districts in Taiwan during 1991–2013. (TIF)

## References

[1] Harrison M. Contagion: how commerce has spread disease. Yale University Press New Haven and London; 2012.

[2] Lounibos LP. Invasions by insect vectors of human disease. Annu Rev Entomol. 2002; 47: 233–66. 10.1146/annurev.ento.47.091201.145206 11729075

[3] Tatem AJ, Rogers DJ, Hay S. Global transport networks and infectious disease spread. Adv Parasitol. 2006; 62: 293–343. 10.1016/S0065-308X(05)62009-X 16647974 PMC3145127

[4] Bryan CS, Moss SW, Kahn RJ. Yellow fever in the Americas. Infect Dis Clin N Am. 2004; 18: 275–292. 10.1016/j.idc.2004.01.007 15145381

[5] Gratz N. Critical review of the vector status of *Aedes albopictus*. Med Vet Entomol. 2004; 18: 215–27. 10.1111/j.0269-283X.2004.00513.x 15347388

[6] Tatem AJ, Hay SI, Rogers DJ. Global traffic and disease vector dispersal. Proc Natl Acad Sci. 2006; 103: 6242–6247. 10.1073/pnas.0508391103 16606847 PMC1435368

[7] Randolph SE, Rogers DJ. The arrival, establishment and spread of exotic diseases: patterns and predictions. Nat Rev Micro. 2010; 8:361–371. 10.1038/nrmicro2336 20372156

[8] Khan IA. Plague: the dreadful visitation occupying the human mind for centuries. Trans R Soc Trop Med Hyg. 2004; 98: 270–277. 10.1016/S0035-9203(03)00059-2 15109549

[9] Kugeler KJ, Staples JE, Hinckley AF, Gage KL, Mead PS. Epidemiology of human plague in the United States, 1900–2012. Emerg Infect Dis. 2015; 21: 16–22. 10.3201/eid2101.140564 25529546 PMC4285253

[10] Smith KF, Carpenter SM. Potential spread of introduced black rat (*Rattus rattus*) parasites to endemic deer mice (*Peromyscus maniculatus*) on the California Channel Islands. Divers Distrib. 2006; 12: 742–748.

[11] Wardrop NA, Fèvre EM, Atkinson PM, Welburn S. The dispersal ecology of Rhodesian sleeping sickness following its introduction to a new area. PLoS Negl Trop Dis. 2013; 7: e2485 10.1371/journal.pntd.0002485 24130913 PMC3794918

[12] Chikeka I, Dumler JS. Neglected bacterial zoonoses. Clin Microbiol Infec. 2015; 21: 404–415. 25964152 10.1016/j.cmi.2015.04.022 PMC4466158

[13] Eisen RJ, Gage KL. Transmission of flea-borne zoonotic agents. Annu Rev Entomol. 2012; 57: 61–82. 10.1146/annurev-ento-120710-100717 21888520

[14] Azad AF. Epidemiology of murine typhus. Annu Rev Entomol. 1990; 35: 553–569. 10.1146/annurev.en.35.010190.003005 2105686

[15] Civen R, Ngo V. Murine typhus: an unrecognized suburban vectorborne disease. Clin Infect Dis. 2008; 46: 913–918. 10.1086/527443 18260783

[16] Blanton LS, Idowu BM, Tatsch TN, Henderson JM, Bouyer DH, Walker DH. Opossums and cat fleas: new insights in the ecology of murine typhus in Galveston, Texas. Am J Trop Med Hyg. 2016; 95: 457–461. 10.4269/ajtmh.16-0197 27273642 PMC4973200

[17] Williams SG, Sacci J, Schriefer M, Andersen E, Fujioka K, Sorvillo F, et al Typhus and typhuslike rickettsiae associated with opossums and their fleas in Los Angeles County, California. J Clin Microbiol. 1992; 30: 1758–1762. 1629332 10.1128/jcm.30.7.1758-1762.1992 PMC265376

[18] Azad AF, Radulovic S, Higgins JA, Noden BH, Troyer JM. Flea-borne rickettsioses: ecologic considerations. Emerg Infect Dis. 1997; 3: 319–327. 10.3201/eid0303.970308 9284376 PMC2627639

[19] Nogueras MM, Pons I, Pla J, Ortuño A, Miret J, Sanfeliu I, et al The role of dogs in the eco-epidemiology of *Rickettsia typhi*, etiological agent of murine typhus. Vet Microbiol. 2013; 163: 97–102. 10.1016/j.vetmic.2012.11.043 23290118

[20] Traub R, Wisseman CL, Farhang-Azad A. The ecology of murine typhus-a critical review. Trop Dis Bull. 1978; 75: 237–317. 705902

[21] Ben Ari T, Neerinckx S, Gage KL, Kreppel K, Laudisoit A, Leirs H, et al Plague and climate: scales matter. PLoS Pathog. 2011; 7: e1002160 10.1371/journal.ppat.1002160 21949648 PMC3174245

[22] Vallée J, Thaojaikong T, Moore CE, Phetsouvanh R, Richards AL, Souris M, et al Contrasting spatial distribution and risk factors for past infection with scrub typhus and murine typhus in Vientiane City, Lao PDR. PLoS Negl Trop Dis. 2010; 4: e909 10.1371/journal.pntd.0000909 21151880 PMC2998433

[23] Yao Z, Tang J, Zhan F. Detection of arbitrarily-shaped clusters using a neighbor-expanding approach: a case study on murine typhus in South Texas. Int J Health Geogr. 2011; 10: 23 10.1186/1476-072X-10-23 21453514 PMC3079590

[24] Lee HC, Ko WC, Lee HL, Chen HY. Clinical manifestations and complications of rickettsiosis in Southern Taiwan. J Formos Med Assoc. 2002; 101: 385–92. 12189643

[25] Chen NY, Huang PY, Leu HS, Chiang PC, Huang CT. Clinical prediction of endemic rickettsioses in northern Taiwan—relevance of peripheral blood atypical lymphocytes. J Microbiol Immunol Infect. 2008; 41: 362–368. 19122916

[26] Lai CH, Huang CK, Weng HC, Chung HC, Liang SH, Lin JN, et al Clinical characteristics of acute Q fever, scrub typhus, and murine typhus with delayed defervescence despite doxycycline treatment. Am J Trop Med Hyg. 2008; 79: 441–446. 18784240

[27] Lai CH, Huang CK, Chen YH, Chang LL, Weng HC, Lin JN, et al Epidemiology of acute Q fever, scrub typhus, and murine typhus, and identification of their clinical characteristics compared to patients with acute febrile illness in Southern Taiwan. J Formos Med Assoc. 2009; 108: 367–376. 10.1016/S0929-6646(09)60080-2 19443290

[28] Chang K, Chen YH, Lee NY, Lee HC, Lin CY, Tsai JJ, et al Murine typhus in southern Taiwan during 1992–2009. Am J Trop Med Hyg. 2012; 87: 141–147. 10.4269/ajtmh.2012.11-0465 22764305 PMC3391039

[29] Courchamp F, Chapuis JL, Pascal M. Mammal invaders on islands: impact, control and control impact. Biol Rev. 2003; 78: 347–383. 14558589 10.1017/s1464793102006061

[30] Moran PAP. Notes on continuous stochastic phenomena. Biometrika 1950; 37: 17–23. 15420245

[31] Anselin L. Local indicators of spatial association—LISA. Geogr Anal. 1995; 27: 93–115.

[32] Anselin L. GeoDa 0.9 user’s guide; 2003 http://geodacenter.org/downloads/pdfs/geoda093.pdf.

[33] Anselin L, Syabri I, Kho Y. GeoDa: an introduction to spatial data analysis. Geogr Anal. 2006; 38: 5–22.

[34] Chang CT, Wang SF, Vadeboncoeur MA, Lin TC. Relating vegetation dynamics to temperature and precipitation at monthly and annual timescales in Taiwan using MODIS vegetation indices. Int J Remote Sens. 2014; 35: 598–620.

[35] European Space Agency. Globcover land cover map. European Space Agency; 2008 (http://geoserver.isciences.com:8080/geonetwork/srv/en/metadata.show?id=228)

[36] Fan MY, Walker DH, Yu SR, Liu QH. Epidemiology and ecology of rickettsial diseases in the People's Republic of China. Rev Infect Dis. 1987; 9: 823–840. 3326129

[37] Lin YR. The spatial structure of ports in Ching Taiwan. Master thesis. Department of History, National Taiwan University, Taipei, Taiwan; 1993 (In Chinese).

[38] Hu W, Tong S, Mengersen K, Oldenburg B. Exploratory spatial analysis of social and environmental factors associated with the incidence of Ross River virus in Brisbane, Australia. Am J Trop Med Hyg. 2007; 76: 814–819. 17488897

[39] Winters AM, Staples JE, Ogen-Odoi A, Mead PS, Griffith K, Owor N, Babi N, Enscore RE, Eisen L, Gage KL. Spatial risk models for human plague in the West Nile region of Uganda. Am J Trop Med Hyg. 2009; 80: 1014–1022. 19478268

[40] Waller LA, Gotway CA. Applied spatial statistics for public health data. John Wiley & Sons, Inc., Hoboken, New Jersey; 2004.

[41] Sartorius B. Modelling determinants, impact, and space–time risk of age-specific mortality in rural South Africa: integrating methods to enhance policy relevance. Glob Health Action. 2013; 6: 19239. 23364094 10.3402/gha.v6i0.19239 PMC3556703

[42] Kery M. Introduction to WinBUGS for ecologists. Academic Press, Burlington; 2010.

[43] Fotheringham AS, Brunsdon C, Charlton M. Geographically weighted regression: the analysis of spatially varying relationships. John Wiley & Sons Chichester, England; 2002.

[44] Chien CH, Chiang PF, Wang HC, Chen KY, Lin MC, Wu HS. Prevalence of ectoparasites and the seroepidemiology of murine typhus in murine-like animals at international ports in Taiwan, 2004–2011. Taiwan Epidemiol Bull. 2012; 28: 320–329.

[45] Kuo CC, Huang JL, Ko CY, Lee PF, Wang HC. Spatial analysis of scrub typhus infection and its association with environmental and socioeconomic factors in Taiwan. Acta Trop. 2011; 120: 52–58. 10.1016/j.actatropica.2011.05.018 21703220

[46] Wardrop NA, Kuo CC, Wang HC, Clements AC, Lee PF, Atkinson PM. Bayesian spatial modelling and the significance of agricultural land use to scrub typhus infection in Taiwan. Geospat Health. 2013; 8: 229–239. 10.4081/gh.2013.69 24258898

[47] Kuo CC, Huang JL, Lin TE, Wang HC. Detection of *Rickettsia* spp. and host and habitat associations of fleas (Siphonaptera) in eastern Taiwan. Med Vet Entomol. 2012; 26: 341–350. 10.1111/j.1365-2915.2012.01009.x 22390200

[48] Kuo CC, Shu PY, Mu JJ, Lee PL, Wu YW, Chung CK, et al Widespread *Rickettsia* spp. infections in ticks (Acari: Ixodoidea) in Taiwan. J Med Entomol. 2015; 52: 1096–1102. 10.1093/jme/tjv083 26336223

[49] Kuo CC, Shu PY, Mu JJ, Wang HC. High prevalence of *Rickettsia* spp. infections in small mammals in Taiwan. Vector-Borne Zoonot. 2015; 15: 13–20. 10.1089/vbz.2014.1584 PMC4307030 25629776

[50] Parola P, Raoult D. Tropical rickettsioses. Clin Dermatol. 2006; 24: 191–200. 10.1016/j.clindermatol.2005.11.007 16714200

[51] Blanton LS, Walker DH. Flea-borne rickettsioses and rickettsiae. Am J Trop Med Hyg. 2017; Epub ahead of print. 10.4269/ajtmh.16-0537 PMC5239709 27799640

[52] Perez-Arellano JL, Fenollar F, Angel-Moreno A, Bolanos M, Hernandez M, Santana E, et al Human *Rickettsia felis* infection, Canary Islands, Spain. Emerg Infect Dis. 2005; 11:1961–1964. 10.3201/eid1112.050711 16485491 PMC3367641

[53] Znazen A, Rolain JM, Hammami A, Jemaa MB, Raoult D. *Rickettsia felis* infection, Tunisia. Emerg Infect Dis. 2006; 12:138–140. 10.3201/eid1201.050876 16494731 PMC3291393

[54] Reif KE, Macaluso KR. Ecology of *Rickettsia felis*: a review. J Med Entomol. 2009; 46:723–736. 19645274 10.1603/033.046.0402 PMC12794530

[55] Yen TT, Hii SF, Graves S, Rees R, Stenos J, Traub RJ. Evidence of exposure to *Rickettsia felis* in Australian patients. One Health. 2016; 2: 95–98. 28616481 10.1016/j.onehlt.2016.06.001 PMC5441329

[56] Fang R, Raoult D. Antigenic classification of *Rickettsia felis* by using monoclonal and polyclonal antibodies. Clin Diagn Lab Immunol. 2003; 10: 221–228. 10.1128/CDLI.10.2.221-228.2003 12626446 PMC150527

[57] Nogueras MM, Cardenosa N, Sanfeliu I, Munoz T, Font B, Segura F. Serological evidence of infection with *Rickettsia typhi* and *Rickettsia felis* among the human population of Catalonia, in the northeast of Spain. Am J Trop Med Hyg. 2006; 74: 123–126. 16407356

[58] Tsai KH, Lu HY, Tsai JJ, Yu SK, Huang JH, Shu PY. Human case of *Rickettsia felis* infection, Taiwan. Emerg Infect Dis. 2008; 14: 1970–1972. 10.3201/eid1412.080515 19046543 PMC2634626

[59] Hsu YM, Lin CC, Chomel BB, Tsai KH, Wu WJ, Huang CG, et al Identification of *Rickettsia felis* in fleas but not ticks on stray cats and dogs and the evidence of *Rickettsia rhipicephali* only in adult stage of *Rhipicephalus sanguineus* and *Rhipicephalus haemaphysaloides*. Comp Immunol Microbiol Infect Dis. 2011; 34: 513–518. 10.1016/j.cimid.2011.09.005 22000945

[60] Tsai KH, Huang CG, Fang CT, Shu PY, Huang JH, Wu WJ. Prevalence of *Rickettsia felis* and the first identification of *Bartonella henselae* Fizz/CAL-1 in cat fleas (Siphonaptera: Pulicidae) from Taiwan. J Med Entomol. 2011; 48: 445–452. 21485388 10.1603/me10022

[61] Lai CH, Chang LL, Lin JN, Tsai KH, Hung YC, Kuo LL, et al Human spotted fever group rickettsioses are underappreciated in southern Taiwan, particularly for the species closely-related to *Rickettsia felis*. PLOS ONE. 2014; 9: e95810 10.1371/journal.pone.0095810 24755560 PMC3995941

[62] Billeter SA, Metzger ME. Limited evidence for *Rickettsia felis* as a cause of zoonotic flea-borne rickettsiosis in southern California. J Med Entomol. 2017; Epub ahead of print. 10.1093/jme/tjw179 28082625

[63] Billeter SA, Diniz PP, Jett LA, Wournell AL, Kjemtrup AM, Padgett KA, Yoshimizu MH, Metzger ME, Barr MC. Detection of *Rickettsia* species in fleas collected from cats in regions endemic and nonendemic for flea-borne rickettsioses in California. Vector-Borne Zoonot. 2016; 16:151–156. 10.1089/vbz.2015.1869 26824189

[64] Labruna MB, Walker DH. *Rickettsia felis* and changing paradigms about pathogenic rickettsiae. Emerg Infect Dis. 2014; 20: 1768–1769. 10.3201/eid2010.131797 25271441 PMC4193273

[65] Guernier V, Lagadec E, LeMinter G, Licciardi S, Balleydier E, Pagès F, et al Fleas of small mammals on Reunion Island: diversity, distribution and epidemiological consequences. PLoS Negl Trop Dis. 2014; 8: e3129 10.1371/journal.pntd.0003129 25188026 PMC4154673

[66] Wang SF, Wang WH, Chang K, Chen YH, Tseng SP, Yen CH, Wu DC, Chen YMA. Severe dengue fever outbreak in Taiwan. Am J Trop Med Hyg. 2016; 94: 193–197. 10.4269/ajtmh.15-0422 26572871 PMC4710429

[67] Jan CS, Elisabet L, Laszlo B, Laura E, My SA, Pasi P, Joacim R. Determinants and drivers of infectious disease threat events in Europe. Emerg Infect Dis. 2016; 22: 581–589. 10.3201/eid2204 26982104 PMC4806948

[68] Wu T, Perrings C, Kinzig A, Collins JP, Minteer BA, Daszak P. Economic growth, urbanization, globalization, and the risks of emerging infectious diseases in China: a review. Ambio. 2017; 46: 18–29. 10.1007/s13280-016-0809-2 27492678 PMC5226902

