# Correction: Significance of major international seaports in the distribution of murine typhus in Taiwan

**DOI:** 10.1371/journal.pntd.0005589

**Published:** 2017-05-03

**Authors:** Chi-Chien Kuo, Nicola Wardrop, Chung-Te Chang, Hsi-Chieh Wang, Peter M. Atkinson

There is an error in reference 55. The correct reference is: Teoh YT, Hii SF, Graves S, Rees R, Stenos J, Traub RJ. Evidence of exposure to *Rickettsia felis* in Australian patients. One Health. 2016; 2: 95–98.
