# Supplementary figures and images for: Significance of major international seaports in the distribution of murine typhus in Taiwan
Source: PLoS Negl Trop Dis. 2017 Mar 6;11(3):e0005430. doi: 10.1371/journal.pntd.0005430 (PMC5354449; doi:10.1371/journal.pntd.0005430)

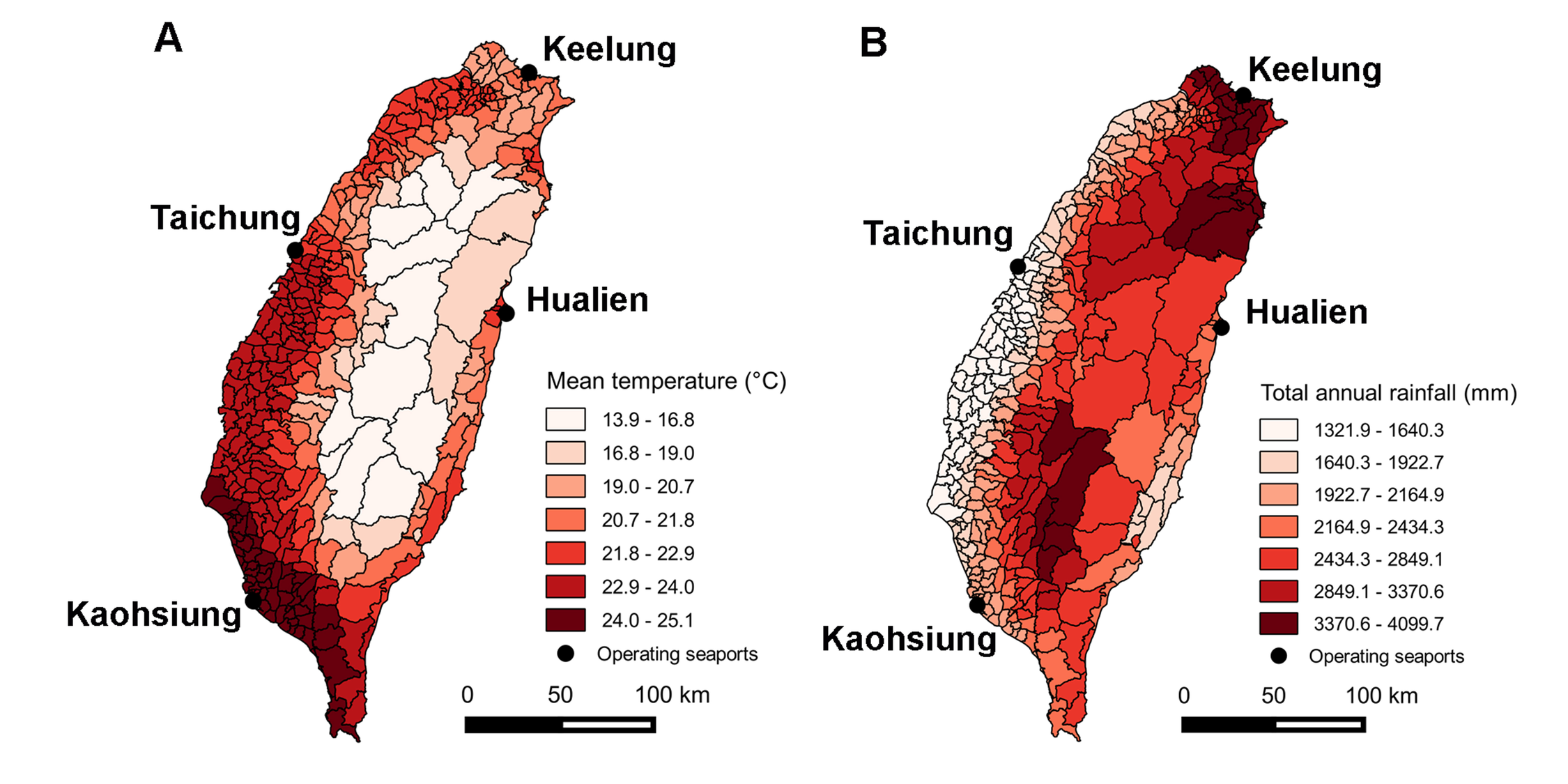

Supplement: S1 Fig — (TIF) [file pntd.0005430.s001.tif]
